# Early Outcome of Multisystem Inflammatory Syndrome in Neonates Diagnosed following Prenatal Maternal COVID-19 Infection: A Three-Case Series

**DOI:** 10.3390/pediatric15040054

**Published:** 2023-10-10

**Authors:** Maria Terciu, Ioana Luca, Emilia Panait, Eugene Leibovitz, Maria Mitrica, Bianca Popovici, Anca Ilea, Oana Gabriela Falup-Pecurariu

**Affiliations:** 1 Children’s Clinical Hospital, 500002 Brasov, Romania; tentiumaria@gmail.com (M.T.); mariamitrica@yahoo.com (M.M.);; 2 Faculty of Medicine, Transilvania University, 500002 Brasov, Romania; 3 Faculty of Health Sciences, Ben-Gurion University of the Negev, Beer-Sheva 84105, Israel

**Keywords:** MIS-N, neonates, COVID-19, SARS-CoV-2

## Abstract

Background: The aim of this case series report is to evaluate the characteristics of multisystem inflammatory syndrome (MIS) in neonates following prenatal maternal COVID-19 infection. Methods: We present a case series of three newborns (≤28 days of age) diagnosed with MIS due to the vertical transmission of SARS-CoV2 infection and admitted from 1 January 2021 to 1 June 2023. The inclusion criteria were negative RT-PCR-SARS-CoV-2 test in infants, initial negative IgM-SARS-CoV-2 in infants followed by the emergence of positive IgG-SARS-CoV-2 antibodies in infants and maternal COVID-19 infection in the third trimester of pregnancy. Patients enrolled in this case series were admitted due to acute febrile illnesses. Results: All three cases occurred in patients born at a mean gestational age of 39 weeks and who were appropriate for gestational age. The mean age at admission was 18.3 days. Fibrinogen (>400 mg/dL) and ferritin (>120 mg/dL) were elevated above the upper normal limit. Elevated levels of myocardial biomarkers (D-dimers, N-terminal pro b-type natriuretic peptide troponin T and creatine phosphokinase myocardial band) were recorded, with normal heart function evaluated using echocardiography. All three patients were treated with antibiotics; one received intravenous immunoglobulin. A 4-week follow-up was completed in two patients when their myocardial biomarkers and ferritin were still elevated but lower compared with previous examinations. D-dimers levels were normalized in 2/3 patients. Conclusions: Subclinical myocarditis was diagnosed as an early outcome in infants with MIS diagnosed postnatally due to the vertical transmission of SARS-CoV2 infection and may represent a new challenge for pediatricians in the pandemic era.

## 1. Introduction

The era of the COVID-19 pandemic has brought a multitude of changes in everyone’s lives and represented a great challenge for healthcare professionals all over the world.

The SARS-CoV-2 virus spreads extremely fast and affects patients of all ages. Even though most children with SARS-CoV-2 infection had asymptomatic or mild forms of the disease, pediatric specialists observed, diagnosed and treated some complications that were gathered under the title of multi-systemic inflammatory syndrome of the child (MIS-C), and this became an exclusion diagnosis in neonatal pathology [1]. It is important to mention that the SARS-CoV-2 virus is linked to release of a cytokine storm, leading to higher risks of thrombosis and increased D-dimers levels [2].

A few case reports implied that MIS in neonates (MIS-N) develops secondary to maternal SARS-CoV-2 infection, unlike MIS-C where SARS-CoV-2 infection and multisystem inflammation occur in the same patient [3]. Infants born to mothers with COVID-19 contracted during pregnancy were shown to develop an early neonatal inflammatory condition [4].

Pregnant women are considered to be a population category that is more vulnerable to COVID-19 infection; therefore, newborns coming from SARS-CoV-2-positive mothers could be more susceptible to SARS-CoV-2 infection by vertical or horizontal transmission [5].

The aim of this case series’ presentation was to describe the clinical and laboratory characteristics of MIS-N in three newborns who were hospitalized at our medical center with this condition and to review the medical literature (at the level of case reports and case series) published in respect to this topic.

## 2. Patients and Methods

Patients enrolled in this case series were admitted at the Children Hospital of Brasov from 1 January 2021 to 1 June 2023 for acute common febrile illnesses. MIS-N was an additional diagnosis, following various laboratory investigations and according to their family history.

The inclusion criteria for MIS-N diagnosis were as follows:

- Negative RT-PCR-SARS-CoV-2 test in infants;
- Negative IgM-SARS-CoV-2 antibodies in infants;
- Positive IgG-SARS-CoV-2 antibodies in infants;
- Maternal COVID-19 infection diagnosed in the third trimester of pregnancy.

None of the mothers was vaccinated against SARS-CoV-2.

The parents of the patients provided their written informed consent for the processing of personal data.

In order to complete this literature review, we used PubMed and Google Scholar as search engines. The keywords used were MISC-N, neonates and COVID-19; newborns; and MIS-C. A total of 17 case reports or case series (57 newborns were included) were retrieved (Supplementary Table S1) [6,7,8,9,10,11,12,13,14,15,16,17,18,19,20,21,22]. The number of patients included in the case series ranged from 2 to 20 newborns.

## 3. Results

Patient no. 1 was a 5-day-old male newborn who presented in the emergency room (ER) with a fever (38.9 °C), drowsiness and apathy that started 24 h before admission (Table 1). He was born through vaginal delivery, at term (39 weeks gestational age), with a normal birth weight and no obstetrical events to mention; he was breastfed.

The mother had a mild form of COVID-19 infection during her last trimester of pregnancy (diagnosed by a positive RT-PCR SARS-CoV-2 test).

**Figure 1:**
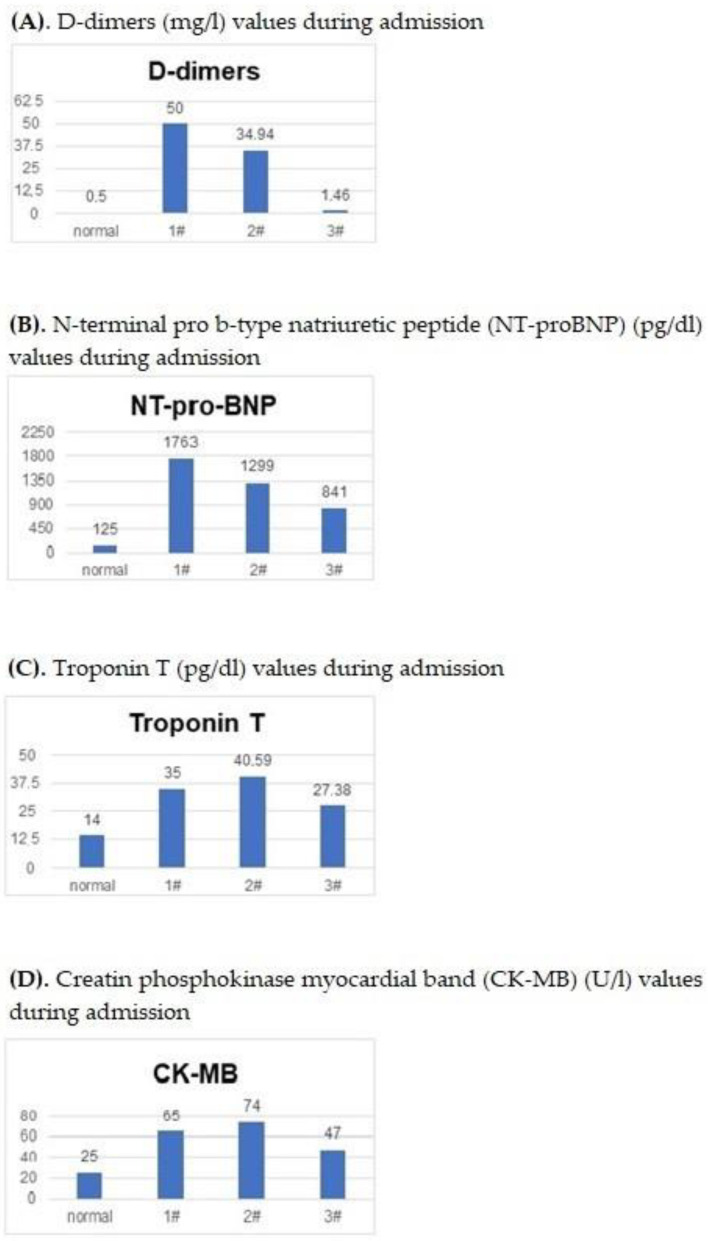
(**A**–**D**). The elevated levels of myocardial biomarkers for each of the three patients.

Blood tests upon admission showed elevated levels of ferritin and fibrinogen as inflammatory markers, with negative blood, urine, stool, nasal and throat swab cultures. He had positive IgG antibodies for the SARS-CoV-2 virus, while the RT-PCR SARS-CoV-2 test and IgM antibodies for the same virus were negative. The complete blood count showed normal leukocytes and platelets values. Myocardial biomarkers were high as well (Figure 1).

Abdominal and transfontanellar ultrasounds showed no mentionable defects. Echocardiography revealed a small patent foramen ovale.

The treatment administered consisted of ampicillin (30 mg/kg four times a day) during his 14 days of hospitalization. He also received corticosteroids and intravenous immunoglobulins. Methylprednisolone was administered at 0.93 mg/kg every 6 h for 2 days, followed by hydrocortisone hemisuccinate 2.5 mg/kg/four times a day for the following 7 days. Intravenous immunoglobulins were administered in the first dose of 0.75 g/kg/day, starting on the ninth day of hospitalization, and a second dose of 0.5 g/kg/day was given on day 11 of admission.

The evolution was favorable; the patient was discharged with no known sequelae, and the follow-up examination (4 weeks later) revealed decreased levels of cardiac biomarkers, except for D-dimer levels, which remained elevated.

The second patient was a 25-day-old female newborn who presented in the ER with bloody diarrheic stools (maximum 13 stools/24 h) starting 7 days before admission, accompanied by a high fever (>39 °C) starting 2 days before presentation.

She was delivered naturally at 39 weeks gestational age, with a normal birth weight and APGAR score. She was fed breast milk and formula.

The mother had a mild form of COVID-19 infection during her last trimester of pregnancy, with diagnosis based on a positive RT-PCR SARS-CoV-2 test.

Blood tests upon admission revealed high levels of ferritin, with negative blood, urine and stool cultures (Table 1). She had negative tests for rotavirus, adenovirus and campylobacter infections. She had positive IgG antibodies for the SARS-CoV-2 virus; the RT-PCR SARS-CoV-2 test and IgM antibodies for the same virus were negative.

The complete blood count of the neonate showed no mentionable modifications. Myocardial biomarkers were elevated (Figure 1).

Abdominal and transfontanellar ultrasounds showed no mentionable defects. Echocardiography revealed a small atrial septal defect.

She received 7 days of intravenous ampicillin—50 mg/kg/day/three times per day. Her clinical evolution was good, and she was discharged after 7 days. Four weeks later, her check-up blood tests showed lower levels of cardiac biomarkers.

The third patient was a 25-day-old female who presented in the ER with cough, rhinorrhea and mild respiratory distress starting 4 days before (Table 1). She was febrile (around 39.5 °C) from 48 h before admission. She was born by caesarian section, at term (39 weeks gestational age), with a normal birth weight (3620 g) and without any obstetrical events, and she was fed formula.

Her mother had a severe form of COVID-19 shortly before antepartum and died 6 days after giving birth.

Blood tests upon admission revealed a slightly elevated C-reactive protein and high levels of ESR and ferritin, with negative blood, urine, stool, nasal and throat swab cultures. The RT-PCR SARS-CoV-2 test and IgM antibodies for the same virus were negative, with positive IgG antibodies.

The complete blood count was normal. She had abnormal levels of NT-pro-BNP, troponin T and CK-MB.

The abdominal ultrasound revealed moderate hydronephrosis.

She received intravenous ampicillin—37.5 mg/kg/four times daily and gentamicin 5 mg/kg once a day for 6 days—and was discharged in good general condition after 7 days of hospitalization.

She did not present for her 4-week follow-up check-up.

Figure 1 presents the graphical display of the elevated values of the myocardial biomarkers (D-dimers—Figure 1A; NT-pro-BNP—Figure 1B; Troponin T—Figure 1C; CK-MB—Figure 1D) found during the hospitalization of the three patients included in this case series presentation compared to values considered normal by the laboratory.

## 4. Discussion

When analyzing the English-language medical literature available on MIS-N, we retrieved 17 case reports or case series describing 57 newborns; of the 17 reports, 13 were from India, while the United States, Saudi Arabia, Brazil and Thailand contributed to the series with 1 case each. In preparing the analysis of these reports, we realized the unexpected predominance of MIS-N cases in India and some other restricted geographic areas, and we were aware that this may be a limitation of our present study.

Of the 57 patients analyzed in this literature search, 6 had a fatal outcome [4,12,13] and 1 underwent limb amputation [9]. Cardiac involvement (left ventricular dysfunction mentioned in six patients), cardiogenic shock, pericardial effusion and pulmonary hemorrhage were present in 12, 2, 2 and 1 reports, respectively. The mortality rates in MIS-N are not known, but the literature reports mortality rates of 1.7–20% for children diagnosed with MIS-C in India, South Africa and Pakistan [20,21,22,23].

When comparing the cases from the literature with our three cases, the similarities in the age group (all of them were <30 days old) were evident. As for the condition at discharge, the majority of them (50/57) were discharged without any known sequelae, similarly to the neonates presented in our study.

Differences may be found in the analyzed relevant literature in respect to the presence of cardiac shock [4,6,10,15], aortic and intra-cardiac thrombosis [9,12], pericardial effusion [11] and coronary artery dilations [4,7]. None of our cases had clinical manifestations of cardiac involvement, and no ultrasound modifications raised the possibility of MISC-N-related myocarditis.

As a matter of fact, the prognosis of children born from COVID-19-infected mothers is considered good, with little chance of long-term consequences [24]. All patients included in our case series had favorable evolution during admission and were discharged in a good general state.

The follow-up examination at 4 weeks after discharge was completed by two of the patients. The newborns were in good general condition, with an upward weight curve. Their myocardial biomarkers and ferritin were still elevated but lower than in previous examinations (Table 1).

Apart from the cardiac involvement, two cases of newborns with erythematous rash were reported in the literature [6,17], while none of our cases presented cutaneous pathologies.

Finally, as seen above, a great number of similarities between the cases were reported, and they only cover a small part of the clinical diversity this syndrome presents, highlighting the need to conduct more studies on this subject and emphasizing the need to screen newborns for MIS-N, although it is considered that the vertical transmission rate of SARS-CoV-2 virus is rather low [25] and that the course of infection for healthy newborns is asymptomatic or characterized by mild clinical manifestations only [26].

## 5. Conclusions

Subclinical myocarditis is one of the early outcomes of multisystem inflammatory syndrome in infants related to prenatal maternal COVID-19 infection, representing a new challenge for pediatricians of the pandemic era.

Further research on this topic is needed, as well as a follow-up of patients in evolution, in order to find out if the COVID-19 infection in pregnant women will have real long-term consequences [27] on children’s health, particularly related to the occurrence of cardiovascular or neurodevelopmental conditions [28].

## Figures and Tables

**Table 1:** Summary of the 3 cases.

Case Number	1	2	3
Admission	April 2022	June 2021	December 2021
Age at admission (days)	5	25	25
Birth	natural	natural	C-section
Gestational age (weeks)	39	39	39
Birth weight (g)	3400	3100	3620
APGAR score	9	9	10
Maternal COVID-19 infection disease severity	mild	mild	Severe (fatal)
Feeding	breastfeeding	mixed feeding	formula
Symptoms onset before admission (days)	1	7	4
Signs and symptoms			
Fever	<3 days	<3 days	<3 days
other	Drowsiness, apathy	bloody diarrheic stools	Cough, rhinorrhea, mild respiratory distress
Inflammation biomarkers			
CRP (mg/dL)	0.48	0.26	2.1
ESR (mm/h)	-	-	50
Fibrinogen (mg/dL)	590	506	-
Ferritin (µg/L)	460	246	174.5
D-dimers (mg/L)	50	34.94	1.46
Myocardial biomarkers			
NT-pro-BNP (pg/dL)	1763	1299	841
Troponin T (pg/dL)	35	40.59	27.38
CK-MB (U/L)	65	74	47
Cultures			
Blood	negative	negative	negative
Urine	negative	negative	negative
Stool	negative	negative	negative
Nasal swab	negative	not performed	negative
Throat swab	negative	not performed	negative
Ultrasound			
Abdominal	normal	normal	hydronephrosis
Echocardiography	patent foramen ovale	atrial septal defect	-
Transfontanellar ultrasound	normal	normal	normal
Treatment			
Intravenous Immunoglobulins	yes	no	no
Antibiotics	ampicillin	ampicillin	ampicillin + gentamicin
Corticosteroids	methylprednisolone; hydrocortisone hemi succinate	no	no
Hospitalization days	14	7	7
Follow-up			
D-dimers (mg/L)	50	0.71	not done
NT-pro-BNP (pg/dL)	322	352	not done
Troponin T (pg/dL)	35	37.25	not done
CK-MB(U/L)	49	not done	not done
CRP (mg/dL)	0.4	0.45	not done
Ferritin (mg/dL)	Not performed	162	not done

## Data Availability

Data is available on request due to privacy and ethical restrictions. The data presented in this study are available on request from the corresponding author. The data are not publicly available due to the confidential nature of personal patients’ information.

## Supplementary Materials

The following supporting information can be downloaded at: https://www.mdpi.com/article/10.3390/pediatric15040054/s1. Table S1. MIS-N in newborns: review of literature.

## Abbreviations

CK-MB	creatine phosphokinase myocardial band
COVID-19	coronavirus disease 2019
CRP	C reactive protein
ER	emergency room
ESR	erythrocyte sedimentation rate
Ig G	immunoglobulin G
Ig M	immunoglobulin M
MIS	multisystem inflammatory syndrome
MIS-C	multisystem inflammatory syndrome in children
MIS-N	multisystem Inflammatory syndrome in neonates
NT-pro-BNP	N-terminal pro b-type natriuretic peptide
RT-PCR	reverse transcription polymerase chain reaction
SARS-CoV-2	severe acute respiratory syndrome coronavirus 2
