# Supplementary material for: Early Outcome of Multisystem Inflammatory Syndrome in Neonates Diagnosed following Prenatal Maternal COVID-19 Infection: A Three-Case Series
Source: Pediatr Rep. 2023 Oct 10;15(4):591–8. doi: 10.3390/pediatric15040054 (PMC10594475; doi:10.3390/pediatric15040054)
Supplement: Supplementary file 1 [file pediatrrep-15-00054-s001.zip › pediatrrep-2534368-SI.pdf]

Supplementary Table 1. MIS-N in newborns: review of literature

Reference<br>Year of publication, country<br>Number of patients	Similarities	Differences
[6]<br>2021, India<br>1 patient	An eight-day-old newborn with elevated NT pro BNP and D-dimers levels. Discharged after treatment with corticoids and immunoglobulins, as 1/3 of our patients received.	The patient in the referenced article had a SARS-COV2 positive test and was admitted for signs of cardiac arrest and pulmonary oedema. Presented also with an erythematous rash.
[7]<br>2021, United States of America<br>1 patient	Neonate with positive IgG and negative IgM anti SARS COV2 antibodies.<br>High levels of NT pro-BNP, D-dimers and troponin. Treated with intravenous immunoglobulin was initiated. The baby recovered after 8 days of hospitalization with no other cardiac sequelae.	The newborn in the referenced article was admitted with left ventricle dysfunction and coronary artery dilation
[4]<br>2021, India<br>20 patients	Seventeen out of 20 enrolled newborns had positive IgG anti SARS COV2, with high levels of D-dimers, NT pro-BNP.<br>They presented bloody stools, like one of our described patients.<br>All of them received treatment with cortisone and immunoglobulins. Eighteen of them made a complete recovery.	Twenty Indian neonates presented with cardiac shock, 8 of them with cardiac injury (left ventricular dysfunction, prolonged QT coronary dilatation)<br>Two died, unlike our patients who recovered with no known sequelae.
[8]<br>2021, Saudi Arabia<br>2 patients	Two cases: one with maternal COVID infection in the third trimester, the second with positive RT-PCR test immediately after birth. One newborn had positive anti SARS CoV-2 IgG antibodies.<br>Both had high levels of CRP, NT pro	Both newborns showed signs of left ventricular dysfunction

	BNP and CK-MB levels. One received antibiotics, and both of them received corticotherapy and immunoglobulins. Both fully recovered.	
[9]<br><br>2021, India<br><br>1 patient	An 11-day-old newborn with positive IgG anti SARS COV2 antibodies, with elevated levels of CRP, D-dimers and NT pro-BNP. Treatment with corticosteroids was administered.	The patient had aortic thrombosis of the right lower limb and limb amputation was necessary.
[10]<br><br>2021, India<br><br>1 patient	A 24 day-old with positive IgG anti SARS CoV-2 IgG antibodies, elevated inflammatory markers. Antibiotic treatment was administered, along with cortisone and immunoglobulins. Documented maternal exposure to SARS CoV-2 virus.	Patient presented with characteristics of cardiogenic shock.
[11]<br><br>2021, Brazil<br><br>1 patient	A newborn of a COVID-19 positive mother who presented with positive anti SARS CoV-2 IgG antibodies. The newborn had elevated D dimers, NT pro-BNP, troponin and inflammatory markers. Treated with antibiotics. Discharged with no known sequelae.	A premature newborn with positive RT-PCR test both for t newborn and his mother). Echocardiography revealed major fetal pericardial effusion.<br><br>Post-partum, the child experienced severe respiratory distress pericardial effusion.
[12]<br><br>2022, India<br><br>3 patients	Known maternal exposure to COVID, both in the first and third trimester.<br><br>Elevated NT pro-BNP, D dimers and troponin levels.<br><br>Positive anti SARS CoV-2 IgG antibodies, negative IgM antibodies. Treated with immunoglobulins and corticotherapy.	Male neonate with severe respiratory distress at birth and cardiac dysfunction.<br><br>Fatal outcome.
[12]<br><br>2022, India<br><br>3 patients	A 6-day-old female with positive anti SARS COV2 IgG antibodies and negative IgM antibodies.<br><br>High levels of D-dimers and T troponin.	The patient developed intra cardiac and aortic thrombosis

	Received intravenous immunoglobulin. Discharged in good condition.	
[13]<br><br>2022, India<br><br>20 patients	Twenty neonates with positive anti SARS COV2 IgG antibodies and negative IgM antibodies. Ten mothers with known maternal exposure.<br><br>Ten patients had elevated NT pro-BNP, 10 had high D-dimers levels, and 1 had high levels of troponin. Nineteen received antibiotics, 17 received corticotherapy and 8 immunoglobulins.	Eight newborns had cardiac dysfunction.<br><br>Two neonates died.
[14]<br><br>2022, India<br><br>1 patient	An 8-day-old neonate presented with diarrheic stools. Positive anti SARS CoV-2 IgG antibodies both in child and mother. High levels of D-dimers and NT pro-BNP. Treated with intravenous immunoglobulin and methylprednisolone. Full recovery.	Developed severe respiratory distress.
[15]<br><br>2021, India<br><br>1 patient	RT-PCR SARS CoV-2 test negative, positive anti SARS COV2 IgG antibodies, high levels of D-dimers and troponin. Mother had positive IgG anti SARS CoV-2 IgG antibodies.<br><br>Baby received intravenous immunoglobulin and 14 days of methylprednisolone and made a full recovery.	A premature newborn with severe respiratory distress and cardiogenic shock shortly after delivery (32 weeks gestation).
[16]<br><br>2021, India<br><br>1 patient	A ten day-old with high fever, positive anti SARS-CoV-2 IgG antibodies and negative RT-PCR test. Elevated levels of CK-MB and troponin T. Treated with immunoglobulins and cortisone.	
[17]<br><br>2021, India	2-day-old patient with known maternal COVID exposure in the first trimester of pregnancy. Positive anti	The patient developed an erythematous rash on face, chest and abdomen, persistent

1 patient	SARS CoV-2 IgG antibodies for child and mother. Elevated levels of D-dimers and NT pro-BNP. Successfully treated with intravenous immunoglobulin and methylprednisolone. Discharged after 11 hospitalization days.	bradycardia and bilious emesis.
[18]<br>2022, India<br>2 patients	Presented with mild respiratory distress), positive anti SARS CoV-2 IgG, positive IgG antibodies in the mother. Elevated levels of NT pro-BNP, D-dimers and troponin T. Successfully discharged after treatment (antibiotics, intravenous immunoglobulin and methylprednisolone).	Severe thrombocytopenia and modified coagulation profile.
[18]<br>2022, India<br>2 patients	Four days-old with poor appetite and lethargy with positive anti SARS CoV-2 IgG antibodies in mother and baby.<br>Elevated D-dimers levels. Treated with intravenous immunoglobulins and systemic steroids	The patient succumbed due to massive pulmonary hemorrhage.
[19]<br>2022, Thailand<br>1 patient	Fifteen-day-old with negative IgM antibodies and RT-PCR SARS COV2 test; mother and child had anti SARS COV2 IgG antibodies. Known maternal exposure to COVID during second trimester of pregnancy.<br>Newborn with elevated NT pro-BNP, troponin and CK-MB levels.<br>Successfully discharged after 12 days of treatment (intravenous immunoglobulin, methylprednisolone, antibiotics).<br>The twin sibling did not develop MIS-N.	Premature baby (33 weeks of gestation) from a twin pregnancy. Echocardiography showed reduced ejection fraction.

Abbreviations

CK-MB - creatine phosphokinase myocardial band

COVID-19 - coronavirus disease 2019

CRP – C reactive protein

Ig G – immunoglobulin G

Ig M – immunoglobulin M

MIS - multisystem inflammatory syndrome

MIS-N - multisystem Inflammatory syndrome in neonates

NT-pro-BNP - N-terminal pro b-type natriuretic peptide

RT-PCR - reverse transcription polymerase chain reaction

SARS-CoV-2 - severe acute respiratory syndrome coronavirus 2
